# Bideposited silver nanocolloid arrays with strong plasmon-induced birefringence for SERS application

**DOI:** 10.1038/s41598-020-77149-0

**Published:** 2020-11-19

**Authors:** Yi-Jun Jen, Wei-Chen Liu, Ming-Yang Cong, Teh-Li Chan

**Affiliations:** Department of Electro-Optical Engineering, National Taipei University of Technology, Taipei, 106 Taiwan

**Keywords:** Nanoscience and technology, Optics and photonics

## Abstract

Silver nano-rod, nano-zigzag, nano-saw, and nano-particle arrays are fabricated with glancing angle bideposition. The structure-dependent anisotropic optical properties of those bideposited nanostructured arrays are measured and investigated. The equivalent birefringence values of nano-rod and nano-zigzag arrays are much larger than crystals found in nature and liquid crystal used in display products. The fact that induced localized plasmon-magnetic field between nanorods dominates the strong phase retardation between p-polarized and s-polarized transmitted wave. For the nano-saw, the strong localized electric field induced between the saw teeth leads to strong SERS signals. Although the bideposited nanoparticles own weak morphological anisotropy, strong optical phase retardation is still detected at wavelengths near 400 nm.

## Introduction

Optical anisotropy is the dependence of the refractive index of a material on the polarization of light. The polarization-dependence of the index causes the double refraction of light in an anisotropic medium. The birefringence is quantified as the maximum difference between the refractive indices of the material. The anisotropy arises from orderly oriented molecules or structures on a subwavelength scale. Solid crystals with anisotropic properties have been used in important optical devices, such as waveplates and beam splitters^1,2^. The anisotropy of liquid crystals, which are widely used in displays, is controlled by an applied electric field^3,4^. Strong birefringence reduces the required thickness for a certain polarization modulation. Recently several metasurfaces are composed of strong birefringent elements to modulate the propagating directions of reflected and transmitted waves. A plasmonic metasurface that is composed of a periodic gold (Au) brick-shape nanoantenna array and a continuous Au film, separated by a silicon dioxide (SiO_2_) dielectric layer functions as a reflective type half waveplate in the near-infrared range^5^. The 340 nm-thick metasurface require an equivalent birefringence larger than 0.5 to offer the phase retardation. A broadband achromatic metalens that consists of GaN-based integrated-resonant unit elements is 800 nm thick to generate phase shift between elements with different optical axis orientations^6^. Actually the element is a half waveplate that can shift the phase of a circular polarized wave by rotating its optical axis. The birefringence needs to be larger than 0.25 to achieve visible imaging.

Most phase retarders used in metasurface or metalens are composed of anisotropic metal-dielectric elements. Such a metal-dielectric anisotropic element has a polarization-dependent complex refractive index whose real part is the index of refraction and whose imaginary part is the extinction coefficient^7^. The effective medium approximation can be used to estimate the optical anisotropy of an anisotropic absorbent composite with a low filling fraction of metal^8^. A high filling fraction of metal induces localized magnetic dipole moments between metal rods, yielding an equivalent permeability that is not unity^9^. A compact plasmonic nanostructure with high equivalent birefringence to generate the required phase retardation is essential in the development of metasurface and metalens.

In this work, glancing angle deposition (GLAD) is used to bideposit serially silver nanozigzag arrays with different pitch lengths. GLAD is a unique form of physical vapor deposition that involves tilting the substrate so that the substrate normal makes an angle θ with respect to the incident vapors^10^. The initial nucleation of condensed adatoms on a substrate induces self-shadowing, preventing the arrival of the vapor flux at the shadowed area. By manipulating the substrate during deposition, various dielectric nanostructures can be mass-produced. In a previous work, titanium dioxide zigzag nanostructured arrays were grown by serial bideposition^11^, in which a tilted substrate was rotated stepwise by half a turn about its normal axis during the evaporation. In bideposition, the substrate is rotated stepwise by half a turn, typically several times during deposition. The birefringence values of bideposited ${\text{TiO}}_{2}$ films with different pitch lengths that were fabricated by serial bideposition were measured and compared. The birefringence of the bideposited thin films was inversely proportional to the pitch length. When the pitch length is similar to or less than the rod width, the individual zigzag structure is shrunk to a column perpendicular to the substrate. The maximum birefringence of a bideposited ${\text{TiO}}_{2}$ thin film with a pitch length of less than 5 nm is 0.15.

Since noble metals have a relatively low melting point and the surface diffusion of metallic adatoms make growing various nanostructures difficult, a slanted silver nanorod array was grown on a smooth substrate using a very low deposition rate and a deposition angle of larger than $80^\circ$^12^. Substrate cooling was recently introduced to reduce the diffusion energy of deposited metal atoms, enabling the growth of various metallic nanostructures, such as nanohelices and nanohooks on a seeded substrate with a feature size as low as 20 nm^13^. Our earlier work showed that gold, silver, and aluminum nanohelix arrays can be successfully grown on a smooth substrate by matching the spin rate to the deposition rate^14^. According to the structure zone model of obliquely deposited silver thin films with a melting temperature of Tm, a columnar structure can be achieved with a $T_{s} /T_{m}$ ratio of less than 0.1 or $0.2 < T_{s} /T_{m} < 0.3$. The silver nanorods become individuated and the rod width falls rapidly as the temperature falls in the range $T_{s} /T_{m} < 0.1$^15^. Therefore, to investigate the minimum pitch length of a zigzag silver structure, liquid nitrogen was used to cool the substrate to the range $T_{s} /T_{m} < 0.1$.

The phase retardations and equivalent birefringence spectra of a slanted nanorod array, a nano-zigzag array, a nanosaw array, and a nanoparticle array were measured and compared with each other. The directions of the principal axes were also verified. GLAD was performed in an electron beam evaporation system to grow various zigzag silver structures. Before deposition, the pressure was pumped to high vacuum pressure of 10^−7^–10^−6^ Torr. The deposition rate was maintained at 0.3 nm/s, as monitored using a quartz crystal microbalance. Ag was deposited on a BK7 glass and Si wafer at a deposition angle of $86^\circ$ from the normal to the substrate over an area of 2.5 × 2.5 cm^2^. During deposition, a liquid nitrogen cooling system was used to control Tsub at about − 140 °C^13,16^.

## Results

**Figure 1:**
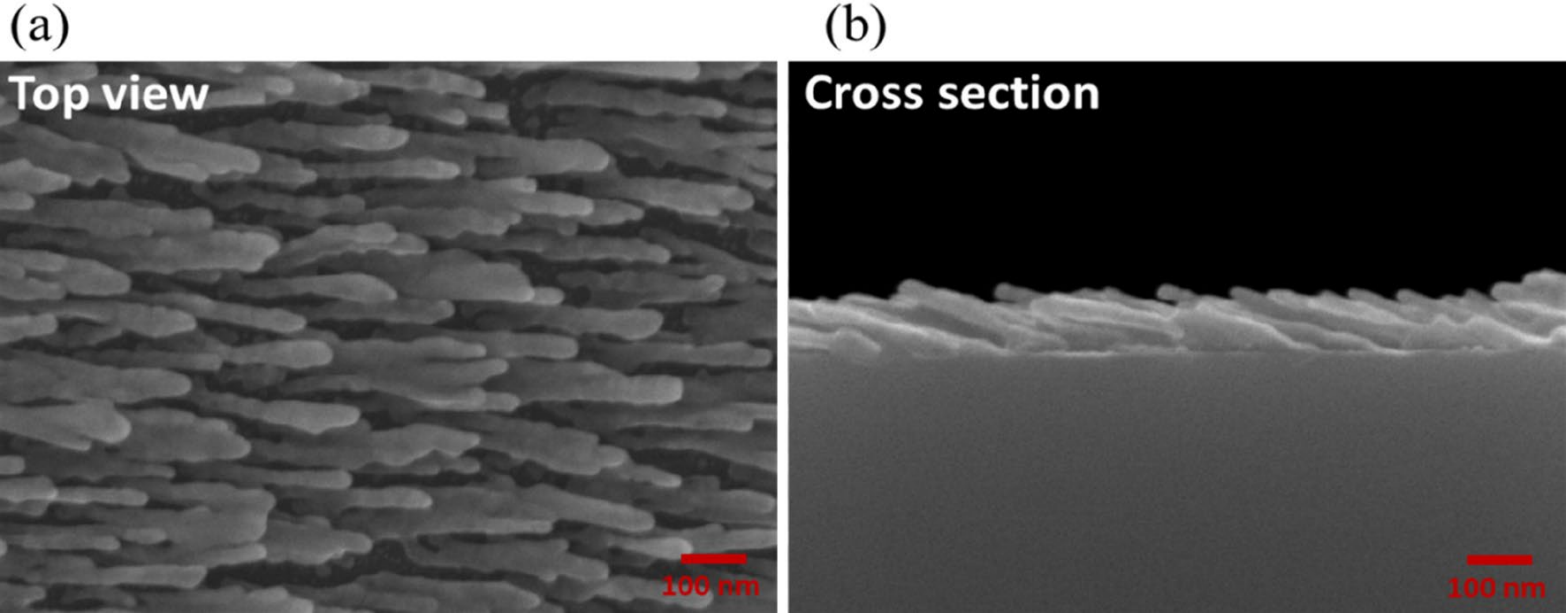
(**a**) Top-view and (**b**) cross-sectional images of silver NRA.

**Figure 2:**
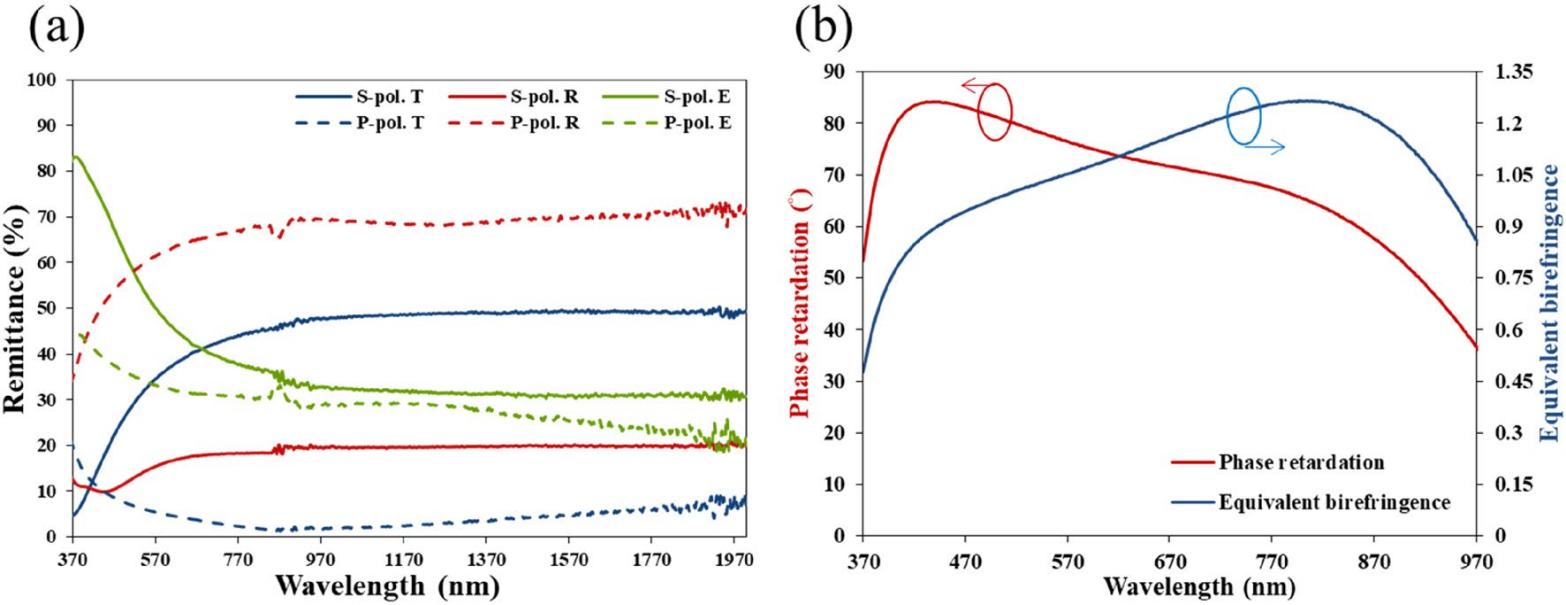
(**a**) P-polarized and s-polarized spectra of reflectance (R), transmittance (T) and extinctance (E) of silver NRA. (**b**) Phase retardation between p-polarized and s-polarized components of wave.

First, a slanted silver nanorod array (NRA) was deposited. Figure 1 shows the cross-sectional and top-view SEM images of the array. The average rod width, D, was 30 nm and the tilt angle ${\beta }$ between the rod and substrate normal was $70^\circ$. The average length of the rods (l) was 251.2 nm. Figure 2a shows the polarization-dependent reflectance (R) and transmittance (T) spectra that were measured using a UV–visible/NIR spectrophotometer (Hitachi High-Tech Corporation, UH4150) at normal incidence. P-polarization and s-polarization refer to the direction of the oscillating electric field- along and perpendicular to the rods, respectively. The extinctance (E) is calculated through the equation E = 1 − R − T^17,18^. When silver nanorods are illuminated by an electromagnetic wave, p-polarized waves induce the longitudinal plasmonic mode and s-polarized waves induce the transverse plasmonic mode^19^. The longitudinal mode of obliquely deposited silver nanorods usually exhibits broadband extinction at wavelengths longer than the resonant wavelengths of the narrow extinction band of the transverse plasmonic mode. The p-polarized reflectance exceeds 60% at wavelengths longer than 546 nm due to the longitudinal plasmon mode resonance in the rods. A commercial spectroscopic ellipsometer (J. A. Woollam Co., VASE) was used to measure the phase retardation between p-polarized and s-polarized components of waves normally incident on the NRA, as shown in Fig. 2b. An incident wave was linearly polarized with oscillation of its electric field at an angle $\varphi$ of $45^\circ$ from the deposition plane (which contains the growth direction of the rods and the surface normal). High phase retardation over $70^\circ$ is distributed over wavelengths from 384 to 714 nm.

For a dielectric birefringent thin film with the phase retardation (PR) that is shown in Fig. 2b. It is equivalent to a birefringence ${\Delta n}_{eq}$ as shown in Fig. 2b.

1 $${\Delta n}_{eq} = \lambda \left( {PR} \right)/\left( {2\pi d} \right)$$

where $\lambda$ and d are the wavelength and thickness of the film, respectively.

The value of ${\Delta n}_{eq}$ exceeds 0.9 from $\lambda = 449\,\,{\text{nm}}$ to $\lambda = 934\,\,{\text{nm}}$. The birefringence reaches its maximum $\Delta n_{eq} =1.278$ at ${\lambda } = 796\,\,\,{\text{nm}}$. The large equivalent birefringence arises from the fact that the equivalent refractive index is negative real for p-polarization and positive real for s-polarization, leading to a large optical path difference between both polarized waves. When the longitudinal plasmonic mode is resonant, the current along the silver nanorods induces a reverse magnetic dipole moment between them. The double negative permittivity and permeability yield a negative index of refraction. The densely packed slanted silver nanorod array has a negative index of refraction for p- polarized light and a positive index of refraction for s-polarized light^20^, which leads to the huge birefringence.

**Figure 3:**
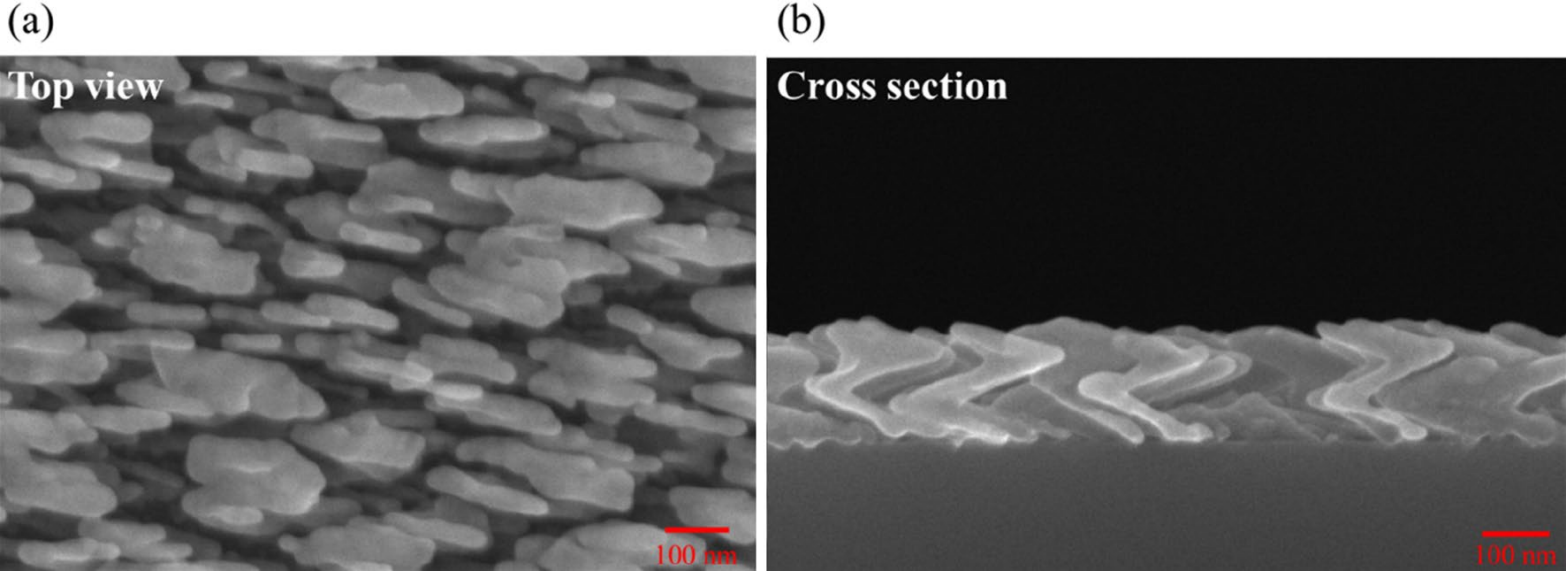
(**a**) Top-view and (**b**) cross-sectional images of silver NZA.

**Figure 4:**
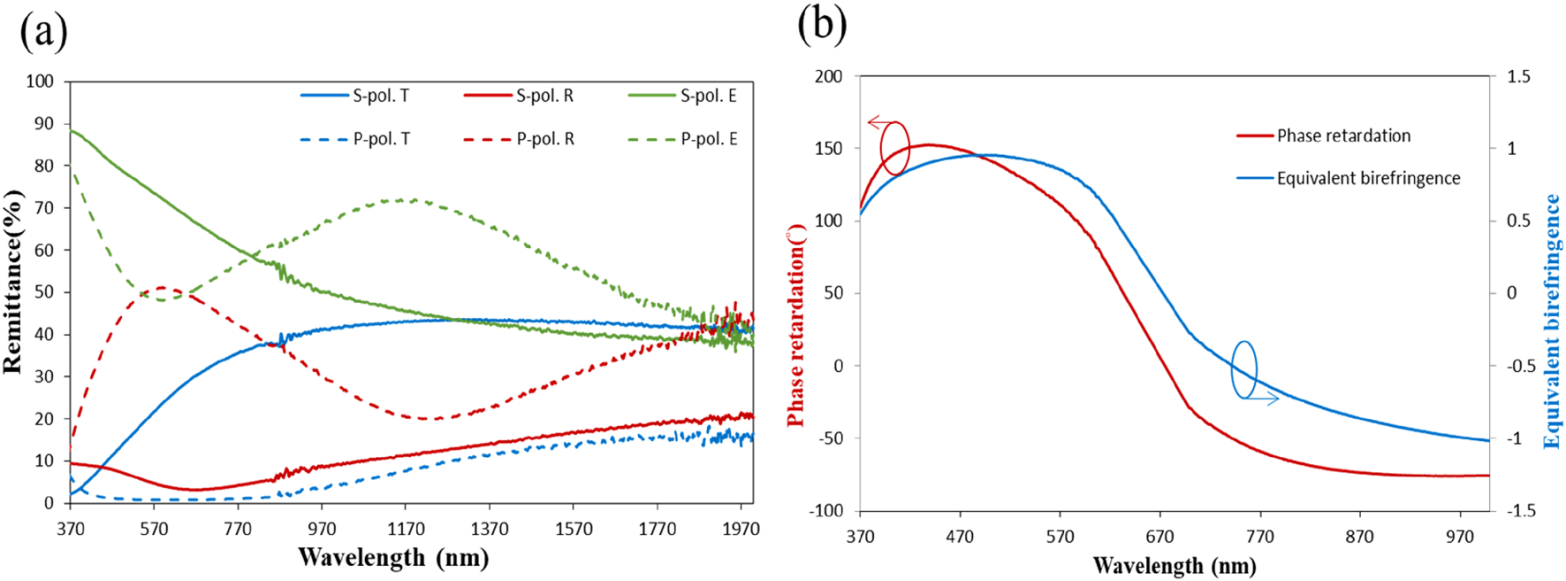
(**a**) P-polarized and s-polarized spectra of reflectance (R), transmittance (T) and extinctance (E) of silver NZA. (**b**) Phase retardation between p-polarized and s-polarized components of wave.

A 1.5-period nano-zigzag array (NZA) with a pitch length of 137.3 nm was deposited at the same deposition angle and deposition rate as the NRA. Figure 3 shows the top-view and cross-sectional SEM images of the ZNA. The average rod width was 26 nm. The tilt angles and rod lengths of the upper, middle and bottom rods were ($\beta , l$) = ($61^\circ$, 162 nm), ($69^\circ$, 112 nm) and ($72^\circ$, 106 nm), respectively. Figure 4a shows the polarization-dependent reflectance and transmittance spectra that were measured at normal incidence. The p-polarized reflectance is lower than that of the aforementioned slanted nanorod array at wavelengths larger than 850nm. The s-polarized transmittance spectrum is similar to that of the Ag NRA. The p-polarized extinctance spectrum shows that a peak occurs at $\lambda = 1193{\text{ nm}}$. The maximum s-polarized extinctance of $88.7{{\% }}$ occurs at the boundary of the spectrum ($\lambda = 370{\text{ nm}}$) and decays to $55.6{{\% }}$ at $\lambda = 863{\text{nm}}$. The strong extinctance between $\lambda = 370\,\,{\text{nm}}$ to s-polarized $\lambda = 863\,\,{\text{ nm}}$ is dominated by transverse plasmonic mode. The maximum phase retardation was $152^\circ$ at $\lambda = 437\,\,{\text{nm}}$, as shown in Fig. 4b. The phase retardation is above $100^\circ$ from $\lambda = 370\,\,{\text{nm}}$ to $\lambda = 587\,\,{\text{nm}}$. The sign of phase retardation changes at a wavelength of 657 nm. The phase retardation rapidly decreases from 0 at $\lambda = 657\,\,{\text{nm}}$ to − 71 at $\lambda = 835\,\,{\text{nm}}$, then smoothly decreases to − 75 at $\lambda = 998\,\,{\text{nm}}$. The positive phase retardation is dominated by transverse plasmonic resonance and the negative phase retardation is dominated by longitudinal plasmonic resonance. Figure 4b plots equivalent birefringence as a function of wavelength. The birefringence reaches its maximum $\Delta {\text{n}}_{eq} = 0.955$ at $\lambda = 489\,\,{\text{nm}}$ and decays to $0.73$ at $\lambda = 597\,\,{\text{nm}}$.

**Figure 5:**
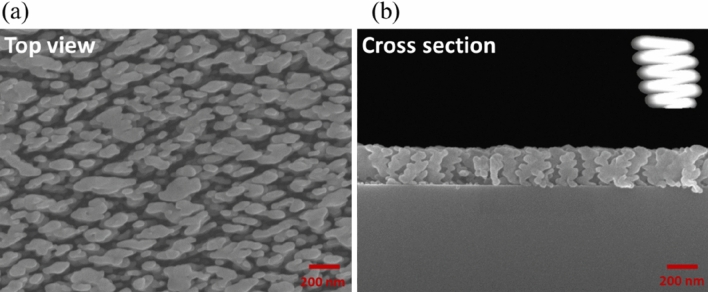
(**a**) Top-view and (**b**) cross-sectional images of silver NSA.

**Figure 6:**
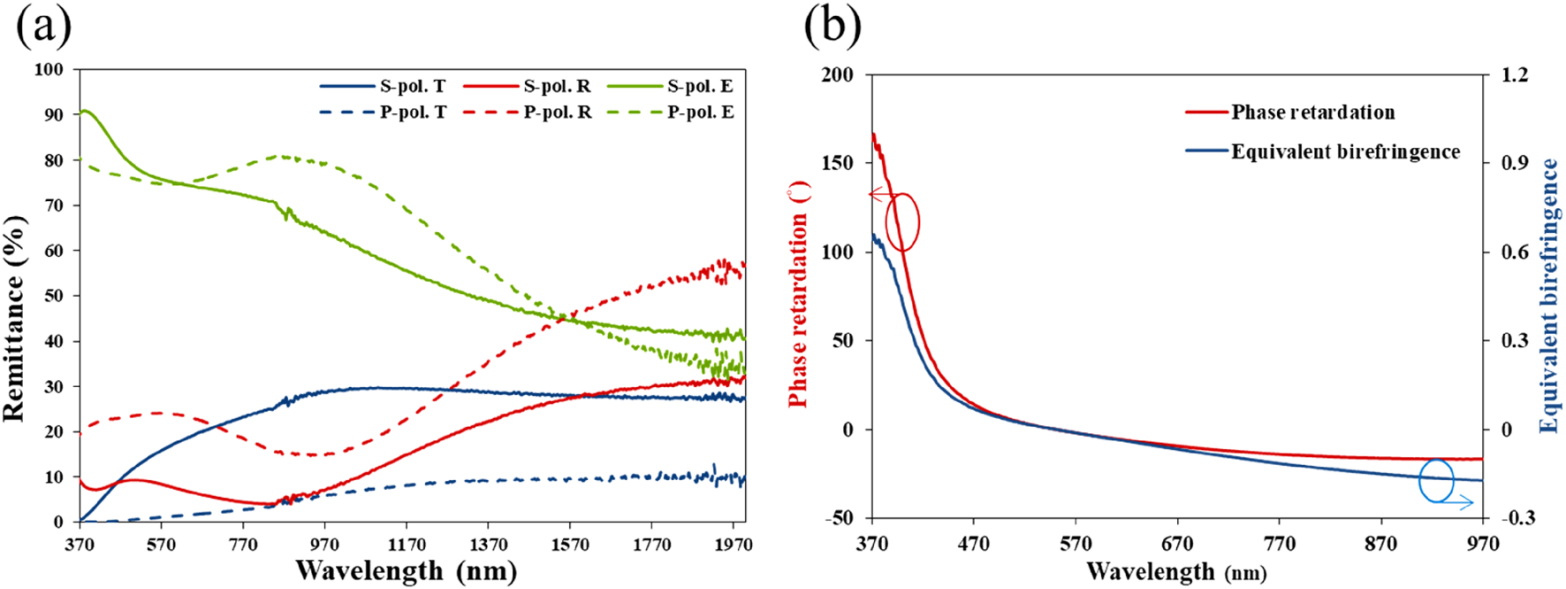
(**a**) P-polarized and s-polarized spectra of reflectance (R), transmittance (T) and extinctance (E) of silver NSA. (**b**) Phase retardation between p-polarized and s-polarized components of wave.

When the pitch length is reduced to 53.7 nm, which is near double the rod width, the spacing between the rods in the zigzag structure disappears and the zigzag structure becomes a saw-like structure. A 4.5 period nanosaw array (NSA) was fabricated and its cross-sectional and top-view SEM images are shown in Fig. 5. A typical nanosaw that was chosen from the SEM is 125 nm-wide and 268 nm-high; each saw tooth is half-circular with a diameter of about 25 nm. Figure 6a shows the polarization-dependent reflectance and transmittance spectra that were measured at normal incidence. Both the p-polarized reflectance and the s-polarized transmittance are lower than those of the NZA and the NRA. The maximum s-polarized extinctance is located at $\lambda = 381\,\,{\text{nm}}$. The extinctance decays rapidly from its maximum to 74.98% at $\lambda = 604\,\,{\text{nm}}$, then decay smoothly to 40.49% at $\lambda = 2000 \,\,{\text{nm}}$. The phase retardation spectrum that was measured at normal incidence reveal a phase retardation peak at a wavelength of 371 nm with a maximum phase retardation of $167^\circ$. The phase retardation decays to half the maximum of its peak at $\lambda =$ 405 nm and changes its sign at a wavelength of 551 nm. At wavelengths from 550 to 1000 nm, the phase retardation decays smoothly from $0.04^\circ$ to $- 16.5^\circ$. Figure 6b plots the equivalent birefringence as a function of wavelength. The maximum birefringence is 0.657 at a wavelength of 372 nm. After the cross-zero wavelength, the birefringence decays to − 0.172 at $\lambda = 970\,\,{\text{nm}}$.

**Figure 7:**
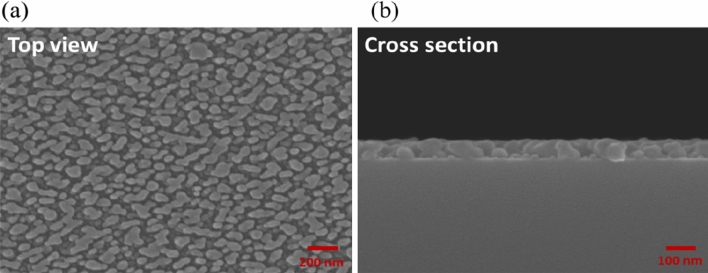
(**a**) Top view and (**b**) cross-sectional view images of silver NPA.

**Figure 8:**
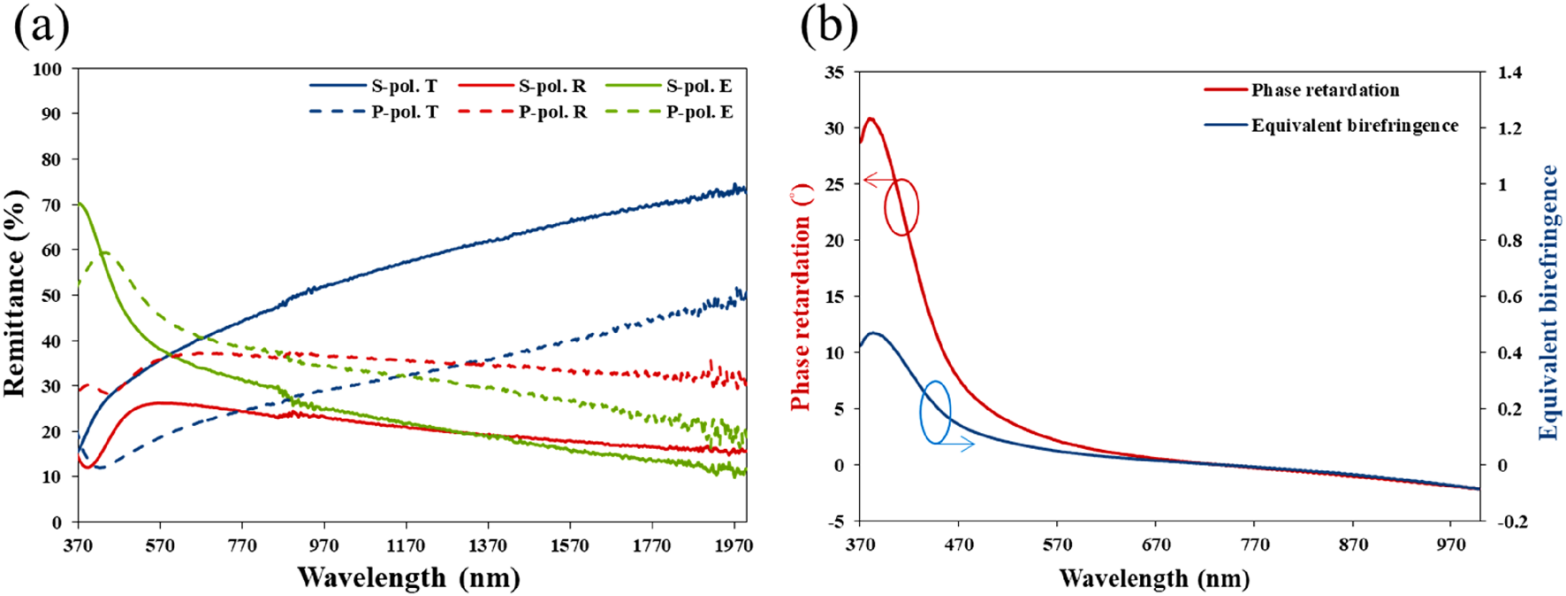
(**a**) P-polarized and s-polarized spectra of reflectance (R), transmittance (T) and extinctance (E) of silver NPA. (**b**) Phase retardation between p-polarized and s-polarized components of wave.

Reducing the pitch further to 42.9 nm changes the nanosaw array into a nanoparticle array (NPA). Figure 7 presents SEM images of a 1.5-period bideposited nanoparticle array. The average particle height is 69.8 nm. The top-view SEM image shows irregularly shaped nanoparticles. Although most of the nanoparticles look like morphological isotropic, the measured reflectance and transmittance spectra and the phase retardation measurements reveal that they exhibit strong optical anisotropy. Figure 8a shows the polarization-dependent reflectance and transmittance spectra of the NPA. The s-polarized transmittance is 21.67% higher than the p-polarized transmittance over wavelengths from 400 to 2000 nm. The p-polarized reflectance is 12.62% higher than the s-polarized reflectance over the whole wavelength range. The maximum s-polarized extinctance is located at ${\lambda } = 373{\text{ nm}}$. The extinctance decays rapidly from its maximum to 38.04% at ${\lambda } = 570\,\,{\text{nm}}$, then decay smoothly to 11.64% at ${\lambda } = 2000\,\,{\text{nm}}$. The measured phase retardation spectra include a phase retardation peak at a wavelength of 380 nm with a maximum phase retardation of $30.85^\circ$. The phase retardation decays to half of its maximum at 434 nm. The sign of phase retardation changes at ${\lambda } = 727\,\,{\text{nm}}$. Figure 8b presents the equivalent birefringence of the 69.8 nm-thick film. The maximum birefringence is 0.468 at a wavelength of 382 nm. After the cross-zero wavelength, the birefringence decays to − 0.074 at λ = 970 nm. The large birefringence values of NSA and NPA near ${\lambda } = 400\,\,{\text{nm}}$ are dominated by the transverse plasmonic mode.

The directions of the equivalent principal axes were verified. The phase retardation spectra of NZA, NSA and NPA were measured again by rotating the sample so that the linear polarization was parallel to the deposition plane ($\varphi = 0^\circ$) or perpendicular to the deposition plane ($\varphi = 90^\circ$). In both cases, the phase retardation of close to zero (less than $4^\circ$) over the wavelengths reveals that the equivalent principal axes for normally incident waves are parallel and perpendicular to the deposition plane.

**Figure 9:**
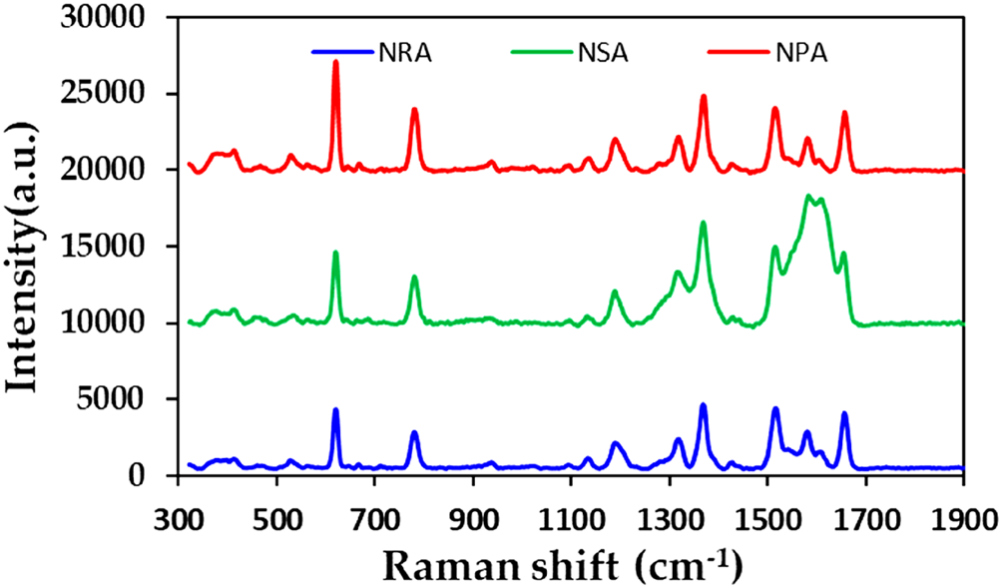
SERS spectra of R6G solution that was adsorbed on NRA, NSA and NPA.

The measured birefringence indicates that the anisotropic nanostructures exist within the deposited array. Since most surface-enhanced Raman scattering (SERS) measurements rely on localized surface plasmon resonance within densely packed metal structures, it inspires us to measure the SERS signals to see whether the nanostructures within the NSA and NPA offer localized enhanced electrical field areas (hot spots) for SERS. The SERS performance of the as-prepared silver NSA and NPA is examined and compared with the NRA that has been demonstrated as a high sensitive SERS substrate^16^. Herein, rhodamine 6G (R6G) was used as a probe molecule. A 4μL droplet of R6G methanol solution with a concentration of $1{\text{ppm}}$ ($2 \times 10^{ - 6}$ M) was dispersed on the surfaces of nanocolloid arrays, which were then dried completely in air. After the droplet had evaporated, the area over which it had spread on each substrate was observed to be approximately $3{\text{ mm}}^{ - 2}$. Raman spectra were obtained using a Confocal Micro RAMAN mapping SYSTEM (Uninanotech co., Ltd, ACRON) with an excitation wavelength of $532{\text{ nm}}$, a power of $300{\text{ mW}}$, and a collection time of $2{\text{ ms}}$. Before saturated adsorption, the SERS signal increased with the concentration of R6G molecules. Figure 9 presents the SERS spectra of R6G solution that was adsorbed on NRA, NSA and NPA that own obviously different morphologies on top of the arrays. The characteristic Raman vibrational peaks of R6G are well resolved at $618$, $778$, $1367$, and $1655{\text{ cm}}^{ - 1}$. The Raman bands at $1655$, $1367$, $778$, and $618{\text{ cm}}^{ - 1}$ are attributed to xanthene ring stretching, ethylamine group wagging, and carbon–oxygen stretching^21^. The intensities of the Raman peaks of the NSA at $1655$ and $1367{\text{ cm}}^{ - 1}$ exceed those of other samples. However, the NPA yields the strongest Raman peaks at $778$, and $618{\text{ cm}}^{ - 1}$. NSA has the most intense characteristic Raman peak intensity of R6G at $1655{\text{ cm}}^{ - 1}$.

The analytical enhancement factor (AEF) was calculated and compared using the formula^22^,

2 $${\text{AEF}} = \frac{{I_{SERS} /C_{SERS} }}{{I_{Ref} /C_{Ref} }}$$

where I_SERS_ and C_SERS_ are the intensity of the SERS spectrum and the concentration of the R6G molecules that were adsorbed on the SERS substrate, respectively, and ${\text{I}}_{{{\text{Ref}}}}$ and ${\text{C}}_{{{\text{Ref}}}}$ are the intensity of the non-SERS spectrum and the concentration of R6G that was adsorbed on the silicon substrate, respectively. The Raman spectrum of R6G with a concentration of $5000\,\,{\text{ppm}}$ ($10^{ - 2}$ M), absorbed on a bare Si surface, was obtained as a reference. The signal-to-noise peaks at specific bands yield the intensity I_SERS_ of R6G. Table 1 provides the AEF values of the three samples for an R6G concentration of $2 \times 10^{ - 6}$ M. The NSA and NPA have higher AEF values than the NRA. The NSA has the characteristic Raman peak intensity of R6G at $1367{\text{ cm}}^{ - 1}$ for the NSA has the largest AEF value of $9.4 \times 10^{4}$. The characteristic peak intensity at $618{\text{ cm}}^{ - 1}$ for the NPA has the largest AEF value of $1.05 \times 10^{5}$. Although the NPA is only $69\,\,{\text{nm}}$ thick, its SERS AEF is higher than that of the NRA.

**Table 1:** AEF of NRA, NSA and NPA with R6G concentration of 1 ppm.

Raman peak (${\text{cm}}^{ - 1}$)	NRA	NSA	NPA
618	$5.76 \times 10^{4}$	$6.90 \times 10^{4}$	$1.05 \times 10^{5}$
778	$3.79 \times 10^{4}$	$4.81 \times 10^{4}$	$6.31 \times 10^{4}$
1367	$6.01 \times 10^{4}$	$9.40 \times 10^{4}$	$6.79 \times 10^{4}$
1655	$6.43 \times 10^{4}$	$7.94 \times 10^{4}$	$6.63 \times 10^{4}$

## Discussion

The high s-polarized extinction of the NSA occurs at wavelengths around 400 nm because the transverse plasmonic mode of a silver rod is excited near the plasmon resonant wavelength. The large phase retardation (equivalent birefringence) occurs comes from that the permittivity of silver that is near minus unity with a small imaginary part near the plasmon resonant wavelength. Therefore, the birefringence of the silver-air composite is very sensitive to the aspect ratio of its nano-structures according to the effective medium approximation.

**Figure 10:**
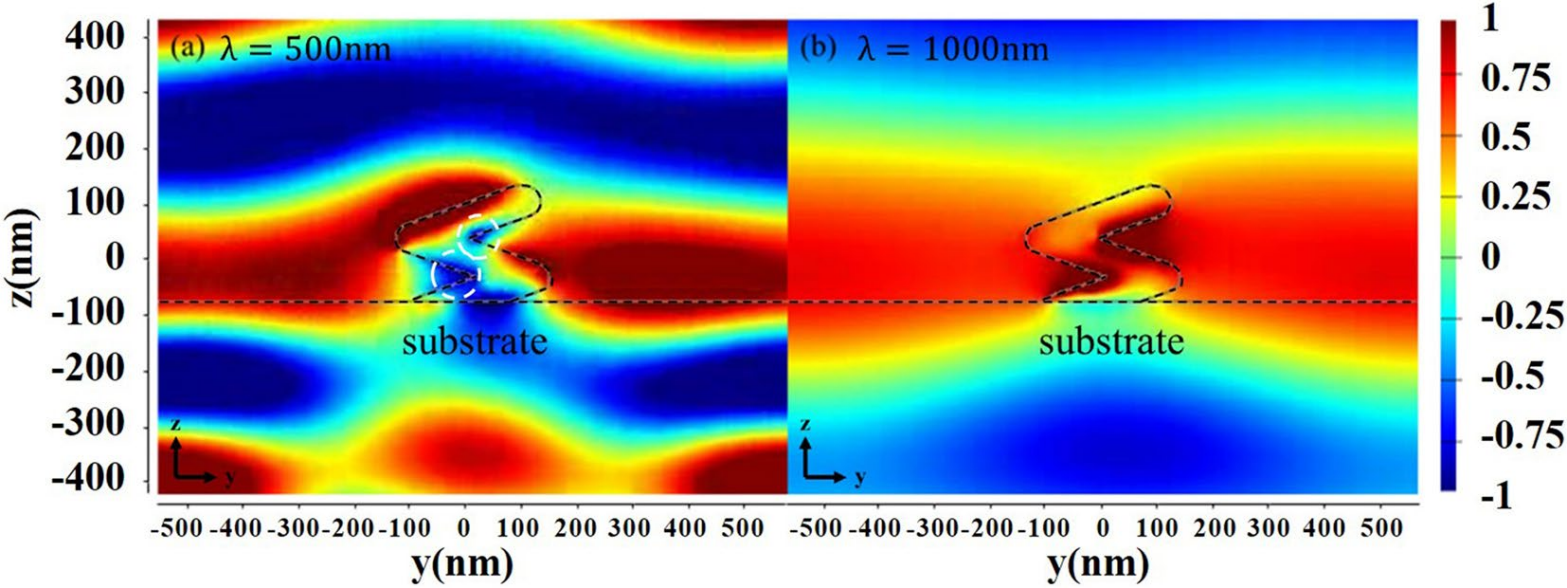
Magnetic field component $H_{x}$ mapped in the yz plane for a nano-zigzag illuminated by p-polarized plane wave at (**a**) $\lambda = 500\,\,{\text{nm}}$ and (**b**) $\lambda = 1000\,\,{\text{nm}}$.

The large phase retardation of the NZA shown in Fig. 4b comes from the fact that a strong magnetic dipole moment is induced between the rods. The finite-difference time-domain (FDTD) (Lumerical FDTD Solutions 8.11.337) simulations involved metallic shapes that were obtained from digitized SEM images of a 300 nm × 300 nm area of experimentally fabricated sample. When the NZA is illuminated with p-polarized incident wave with a wavelength of 500 nm, the magnetic field distribution on the cross-section of a nano-zigzag is shown in Fig. 10a. The reversal magnetic field is then observed between the rods, which can explain the large phase retardation of the NZA. The simulation of wave propagating through a nano-zigzag is to qualitatively explain the birefringence enhancement. The magnetic field near the junction of rods is reversed with respective to the magnetic field of wave propagating at the same attitude. The fact that birefringence over unity is unable to be estimated with effective medium approximation because the equivalent permeability has been departed from unity. It is worth to investigate the localized magnetic field within the nanostructure. The magnetic field reversed areas are marked with white dash lines in Fig. 10. However, the magnetic field between rods is enhanced and in the same direction as the magnetic field of wave propagating in the air at $\lambda = 1000\,\,{\text{nm}}$ as shown in Fig. 10b. The p-polarized electric field propagation phenomena at ${\lambda } = 400\,\,{\text{nm}}$ and ${\lambda } = 1000\,\,{\text{nm}}$ are shown in Fig. S2 to show the different signs of phase retardation at the two wavelengths. At ${\lambda } = 400\,\,{\text{nm}}$, the wave front of the transmitted p-polarized wave advances that of s-polarized wave. However, the wave front of the transmitted s-polarized wave advances that of p-polarized wave at ${\lambda } = 1000\,\,{\text{nm}}$.

For the NSA, the magnitude of equivalent birefringence at wavelengths over the cross-zero wavelength is reduced and smaller than that of the NZA. The spacing between rods is shrunk and disappear for the NSA and the magnetic field enhancement phenomenon is eliminated. Therefore, only large phase retardation exists at short wavelengths around 400 nm. As shown in Fig. S3, the aforementioned nanosaw is adopted here to simulate the electric field intensity for an incident wave with unitary amplitude. Figure S4 plot electric field intensity distributions on a logarithmic scale in the cross-section of a typical nanosaw at four wavelengths of 400 nm, 600 nm, 1000 nm and 1500 nm. The electric field is enhanced and localized between the saw teeth, called hot spots. The p-polarized illumination leads to strong enhancement of electric field and the strongest local field enhancement occurs at ${\lambda } = 600\,\,{\text{nm}}$ that is close to the excitation wavelength of light source used in SERS measurement. The strong SERS signal of the NSA is then understood by analyzing the electric field enhancement.

In conclusion, using the cryogenic GLAD method, silver nanostructures with feature sizes around 25 nm were fabricated. The birefringence magnitudes of NRA and NZA exceed unity at certain wavelength ranges. By suppressing the pitch length of a silver zigzag array become saw-like or irregular particle arrays. The optical anisotropy does not disappear as the morphology of the silver colloids is near isotropic. For a 69 nm-thick nano-particle array, the phase retardation is $30^\circ$ at a wavelength of 375 nm. Although the morphology of the NP is near isotropic, an optical axis exists along the deposition plane. The localized transverse plasmon mode exhibits birefringence around resonant wavelengths of around 400 nm. The maximum birefringence values of nanorod array (1.278), nano-zigzag array (0.955), nano-saw array (0.657) and nano-particle array (0.468) exceed the birefringence values of calcite, Rutile, and liquid crystal. It is demonstrated that bideposited silver particles have a hidden anisotropic structure. The bideposition can achieve by GLAD co-sputtering with two different targets. Various birefringence spectra will be expected for various nanostructures grown with diverse deposition method^21,22^.

In this work, the parameter we varied for a bideposited silver nanocolloid array is the period of bideposition. It is worth to investigate the birefringence values of silver nanocolloid arrays with same bideposited periods but different period numbers (thicknesses) in order to demonstrate the factors that lead to huge birefringence experimentally. However, the tilt angle and rod width of an obliquely deposited array are varied with increasing thickness. The phenomenon that the rod width increasing with thickness in GLAD is called “fan-out”^23^. It is difficult to maintain the same structure for each bideposited period within a multiple period array. It has been shown that using phi-sweep technique^24^ can improve the fan-out phenomena for dielectric nanorod arrays. Whether the phi-sweep technique is suitable to improve the fan-out phenomena of metal nanorod arrays needs to be investigated in the future.

The method of fabrication that is proposed herein can be used to fabricate various ultra-small optical anisotropic nanocolloids. The positive or negative birefringence at certain wavelengths can be achieved by sculpturing a suitable shape of nanostructure. By arranging the proposed nanostructures on a periodic seeded surface, the high birefringence will reduce the thickness requirement for polarization and phase modulation and the transmission can be increased function as a high efficient waveplate. The deposited plasmonic nanocolloids with high anisotropic property could be efficient unit elements for a metasurface or metalens to manipulate the light wave with ultra-thin thicknesses. The effect of plasmonic resonance within the hidden anisotropic structure on SERS measurement reveals that the NSA and NPA have excellent sensitivity and high AEF values in the detection of ultra-low concentrations of R6G. The NPA is demonstrated as a metasurface to show higher SERS signal than the NRA. Therefore, this work introduces the nanostructure-dependent plasmonic birefringence for applications in metasurfaces and bio-applications.

## Supplementary information

Supplementary Information
