# Supplementary material for: Bideposited silver nanocolloid arrays with strong plasmon-induced birefringence for SERS application
Source: Sci Rep. 2020 Nov 19;10:20143. doi: 10.1038/s41598-020-77149-0 (PMC7678831; doi:10.1038/s41598-020-77149-0)
Supplement: Supplementary file 1 — Supplementary Information [file 41598_2020_77149_MOESM1_ESM.docx]

**Supplementary Information**

**Bideposited silver nanocolloid arrays with strong plasmon-induced birefringence for SERS application**

Yi-Jun Jen ^1,^*, Wei-Chen Liu ^1^, Ming-Yang Cong ^1^, Teh-Li Chan ^1^

^1^Department of Electro-Optical Engineering, National Taipei University of Technology, Taipei 106, Taiwan

*****Correspondence: jyjun@ntut.edu.tw


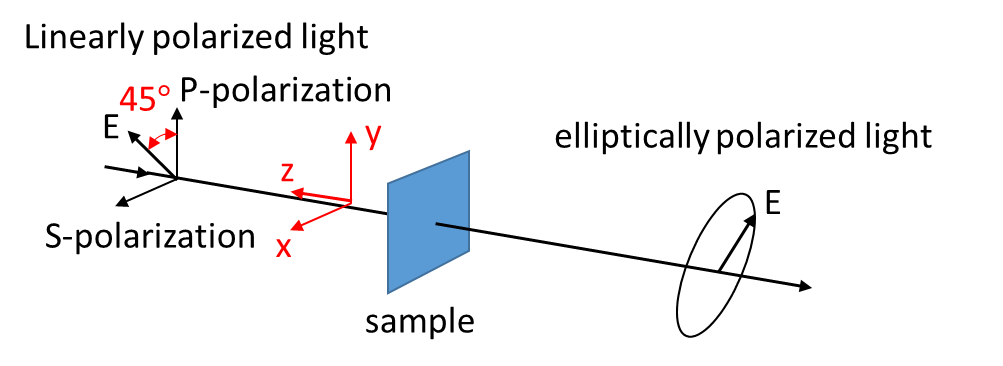


Figure S1. Interaction of polarized light with a sample; z-y plane is the deposition plane


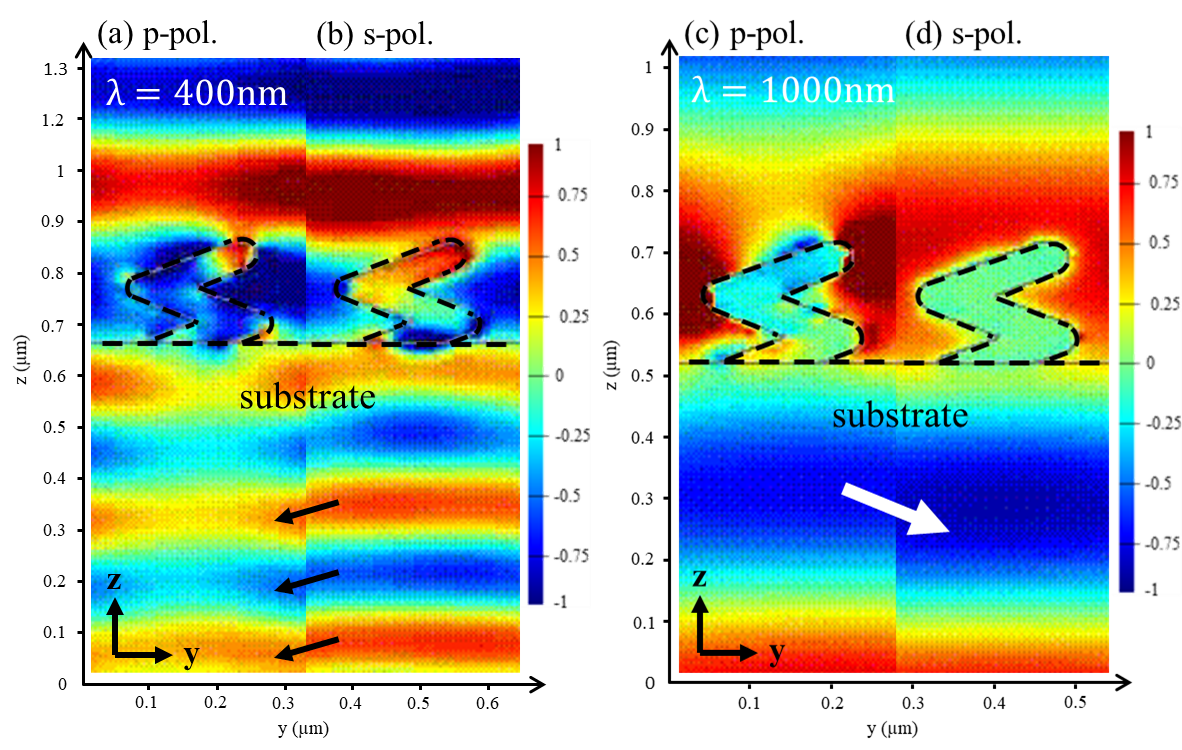


Figure S2. Propagation phenomena of the p-polarized electric field component $E_{y}$ mapped in the yz plane for $\left[ a,c \right]$ a nano-zigzag illuminated by a p-polarized plane wave (electric field parallel to the y axis), when $\lambda\in\{400,1000\}$ nm; the s-polarizaed electric field component $E_{x}$ mapped in the yz plane for $\left[ b,d \right]$ a NZA illuminated by a s-polarized plane wave (electric field parallel to the x axis) , when $\lambda\in\{400,1000\}$ nm.


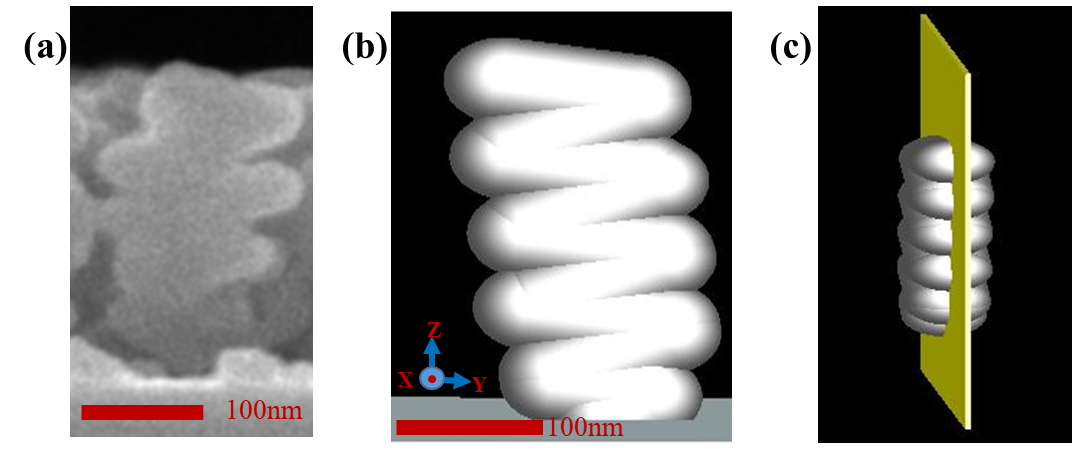


Figure S3. (a) The cross section-view SEM image of a typical nano-saw; (b) The schematic drawing of a nano-saw for simulation; (c) The monitor is placed in the middle of the nano-saw.


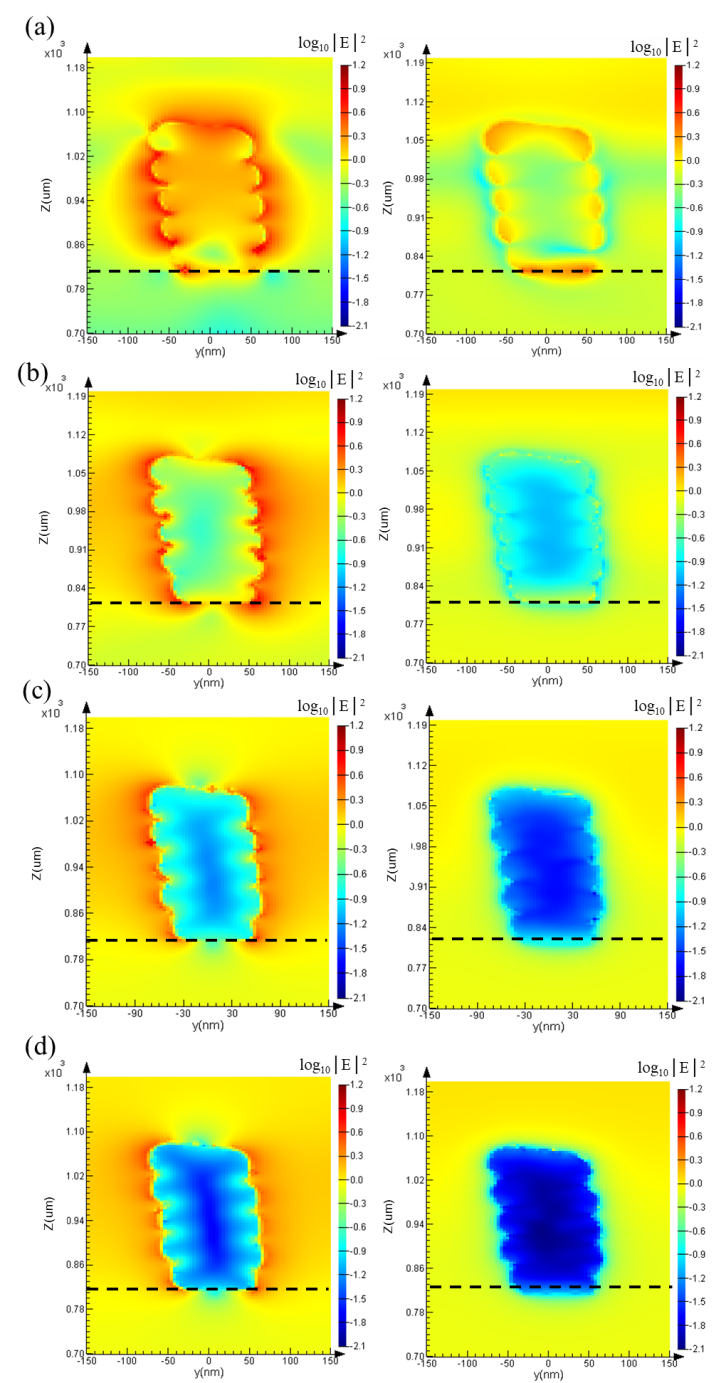


Figure S4. The electric field intensity diagrams simulated at wavelengths of (a) 400 nm, (b) 600 nm, (c) 1000 nm, (d) 1500 nm.


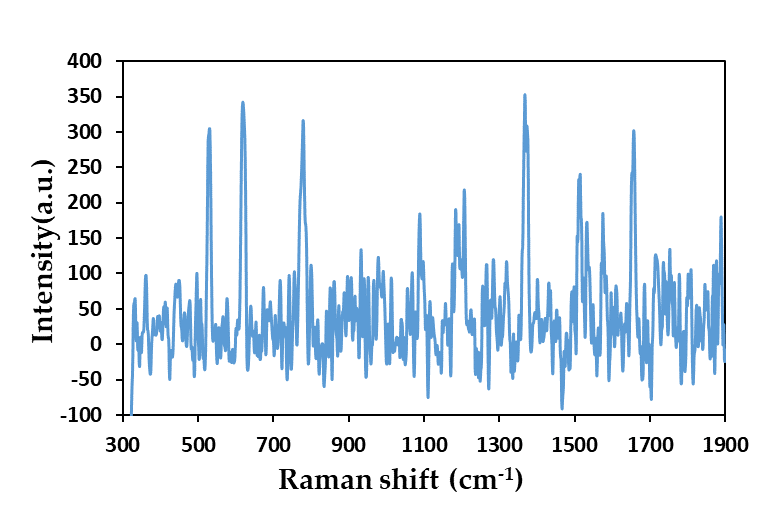


Figure S5. Reference Raman spectrum of R6G with a concentration of $5000ppm$
